# Sciatic and Posterior Femoral Cutaneous Nerve Anomalies and Their Clinical Implications for Sciatica and Piriformis Syndrome: A Cadaveric Case Report

**DOI:** 10.7759/cureus.106901

**Published:** 2026-04-12

**Authors:** Camille Reaux, Sara G Teel, Nicholas C Heppner-Lundin, Zachary Norrod, Ahmad Imam, Kamal A Abouzaid

**Affiliations:** 1 College of Medicine, William Carey University College of Osteopathic Medicine, Hattiesburg, USA; 2 Department of Anatomical Sciences, William Carey University College of Osteopathic Medicine, Hattiesburg, USA; 3 Department of Anatomy, William Carey University College of Osteopathic Medicine, Hattiesburg, USA

**Keywords:** cadaveric case report, common fibular nerve, piriformis, piriformis syndrome, sciatica, sciatic nerve anomaly, tibial nerve

## Abstract

The sciatic nerve (SN), the largest nerve in the human body, normally exits the pelvis inferior to the piriformis muscle (PM) before dividing into its two terminal branches, the tibial nerve (TN) and the common fibular nerve (CFN). Variations in the anatomy of the SN and the posterior femoral cutaneous nerve (PFCN) may occur during embryologic development and can have important clinical implications, including piriformis syndrome, sciatica, and increased susceptibility to iatrogenic injury. This case report describes a rare concurrent variation of both the SN and PFCN identified during routine cadaveric dissection. During a pedagogical dissection of the gluteal region in an 84-year-old female cadaver, notable anomalies in the formation and course of these nerves were observed. Instead of emerging as a single trunk, the CFN and TN exited the pelvis independently through the greater sciatic foramen. The CFN pierced the PM, dividing it into two muscular heads before converging with the TN approximately one inch inferior to the muscle to form a single SN trunk. The TN exited inferior to the PM and followed a relatively typical course. Additionally, the PFCN exited the pelvis as separate nerve roots, with the most lateral root piercing the PM while the remaining roots passed inferior to the muscle. These findings correspond to a unilateral type 2(B) variation of the SN according to the Beaton and Anson classification. Anatomical variations of the SN and PFCN are clinically significant because they may predispose individuals to nerve entrapment syndromes such as piriformis syndrome and may increase the risk of nerve injury during procedures, including hip arthroplasty, gluteal injections, and proximal hamstring repair. Recognition and documentation of these variants are essential for improving diagnostic accuracy, guiding surgical planning, and supporting more individualized approaches to patient care.

## Introduction

The sciatic nerve (SN), the body’s largest nerve, originates from the anterior rami of the lumbosacral trunk (LST) and runs through the posterior pelvis, where it converges with sacral nerves S1-S3 to form a thick continuous nerve [1]. Beginning its course in the pelvis, it runs through the gluteal region to enter the posterior compartment of the thigh, where it innervates all muscles in this area. The SN also innervates muscles below the knee after dividing into its two terminal branches, the common fibular nerve (CFN) and the tibial nerve (TN) at the apex of the popliteal fossa. The TN is made up of the L4-S3 nerve roots, and the CFN is made up of contributions from the LST (L4-L5) and S1-S2 nerve roots.

The posterior femoral cutaneous nerve (PFCN) is derived from the posterior division of the anterior sacral rami of S1 and S2 and from the anterior division of the anterior rami of S2 and S3. Its course is similar to the SN in that it exits the pelvis by passing through the greater sciatic foramen and courses underneath the piriformis muscle (PM). It provides sensory innervation to the inferior buttock, part of the scrotum or labium majus, the back of the thigh, and the popliteal fossa [2].

Anatomical variations of the SN and PFCN can occur during embryological development. Like all spinal nerves, these nerves originate from neuroepithelial cells in the developing spinal cord around week 4 of embryologic growth. As the myotome branches of the SN and PFCN develop from somites, they simultaneously receive innervation, creating each respective nerve [3].

The anatomy of the PM plays an important role in classifying SN anomalies [4]. This muscle originates from the sacrum and sacrotuberous ligament and inserts into the greater trochanter of the femur. Innervated by the nerve to piriformis (S1-S2) and receiving multiple arterial supplies (superior and inferior gluteal arteries, internal pudendal artery, and lateral sacral arteries), this muscle is a common cause of back pain [5].

Based on the classification of SN variations outlined by Beaton and Anson [4], this case is identified as a type 2(B) variation. A review conducted in 2010 reported that this variation accounts 80.9% of variant gluteal regions [6]. Beaton and Anson identified six anatomical classifications of the SN in relation to the PM. In our case, the CFN has its typical contributions, but it exits the pelvis independently of the TN, piercing the piriformis to enter the posterior compartment of the thigh rather than passing underneath this muscle. The TN exits the pelvis independently but follows its typical course in passing underneath the PM. Due to its size, contributions, and location, the SN is associated with common clinical complaints, including piriformis syndrome (PS) [6] and iatrogenic injury due to liposuction or total hip arthroplasty [7,8]. Specifically, type 2 SN variations are associated with higher rates of symptomatic sciatica compared to other variations [9]. Our donor’s PFCN was also anomalous. The PFCN’s contributories remain anatomically typical, but its course is variant. Two of the roots exit the pelvis inferior to the PM, whereas the most lateral root pierces the PM just medial to the CFN. Publishing SN and PFCN variations provides more accurate data on the prevalence of nervous system anomalies, enabling clinicians and surgeons more clarity in their practices.

In this case study, we evaluate the SN and PFCN anomalies found during normal dissection of a human cadaver at William Carey University College of Osteopathic Medicine. Furthermore, we will discuss the clinical manifestations of SN and PFCN neuropathy and how the abnormal anatomy of these nerves correlates to patient well-being.

## Case presentation

During a pedagogical cadaveric dissection of the gluteal region conducted by first-year medical students, unusual variations in the course and formation of the SN and the PFCN were identified in an 84-year-old female donor. The donor was received through the Southern Alabama Anatomical Gift Program, and the listed cause of death on the death certificate was malnutrition.

**Figure 1:**
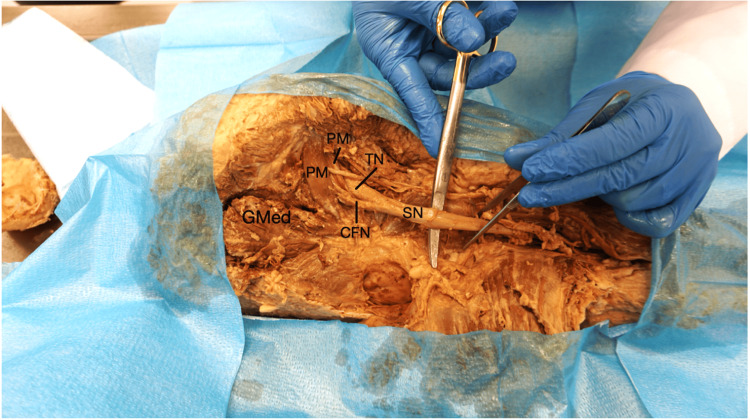
Sciatic nerve (SN) anomaly View of the gluteal region showing the PM is split into two heads by the separation of the SN into the CFN and the TN before converging inferior to the PM. The GMed is labeled for anatomical reference. PM: piriformis muscle, TN: tibial nerve, CFN: common fibular nerve, GMed: gluteus medius

Dissection of the left gluteal region revealed two notable anomalies following reflection of the gluteus maximus. Rather than emerging as a unified SN, the CFN and TN exited the pelvis separately through the greater sciatic foramen. The CFN pierced the PM, dividing it into two distinct muscular heads (Figure 1).

**Figure 2:**
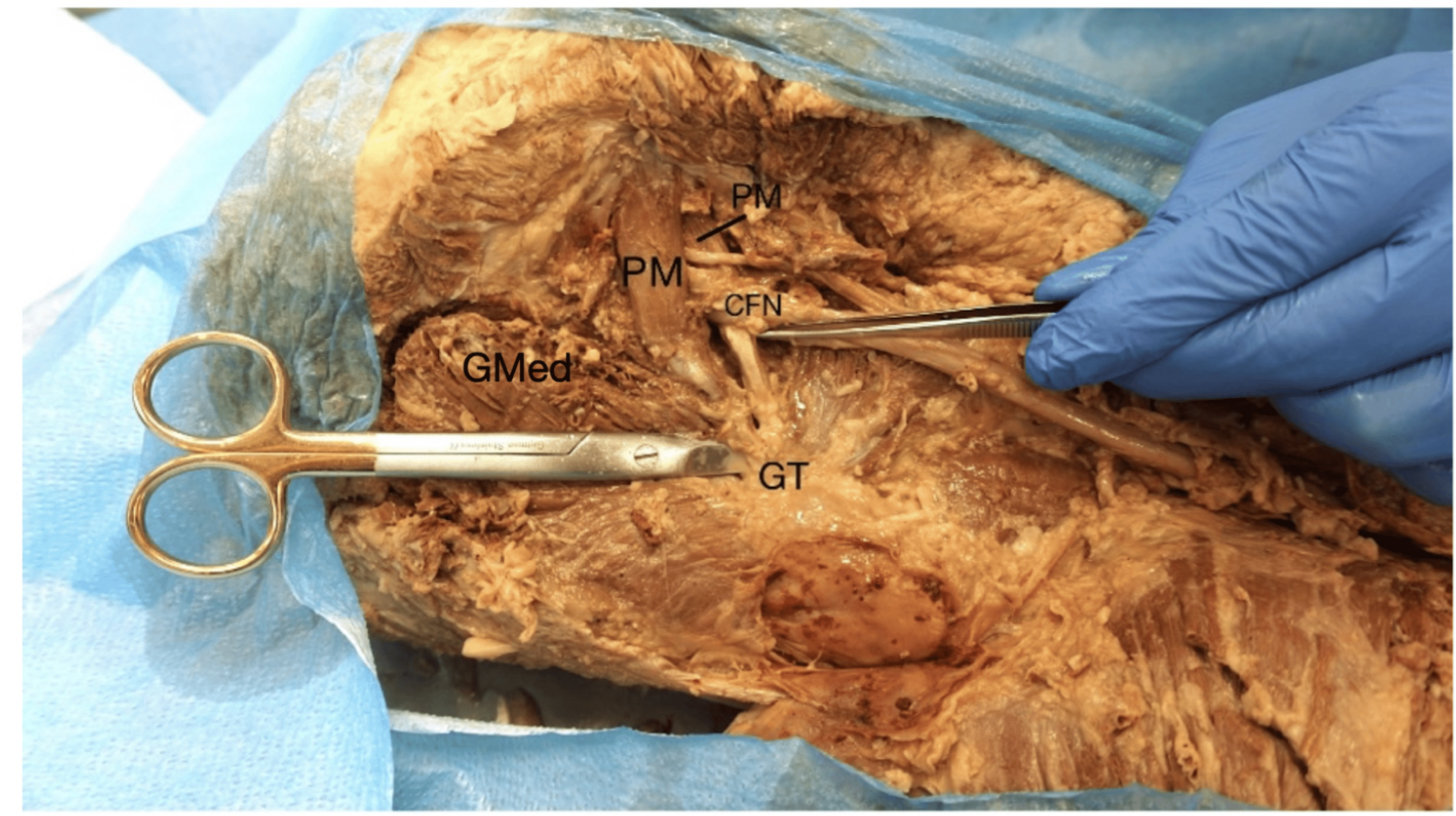
Common tendinous insertion of PM The PM is split into two heads by the CFN but has one insertion on the superior border of the greater trochanter of the femur. Fibers from the GMed join the PM tendon. PM: piriformis muscle, CFN: common fibular nerve, GT: greater trochanter, GMed: gluteus medius

These heads subsequently rejoined as they exited the greater sciatic foramen, forming a common tendon that inserted onto the medial surface of the greater trochanter of the femur. In addition, fibers from the gluteus medius muscle were observed contributing to the piriformis tendon prior to its insertion on the greater trochanter. The TN exited the greater sciatic foramen inferior to the PM. Approximately one inch inferior to the piriformis, the CFN and TN converged within the gluteal region to form a single SN trunk (Figure 2).

**Figure 3:**
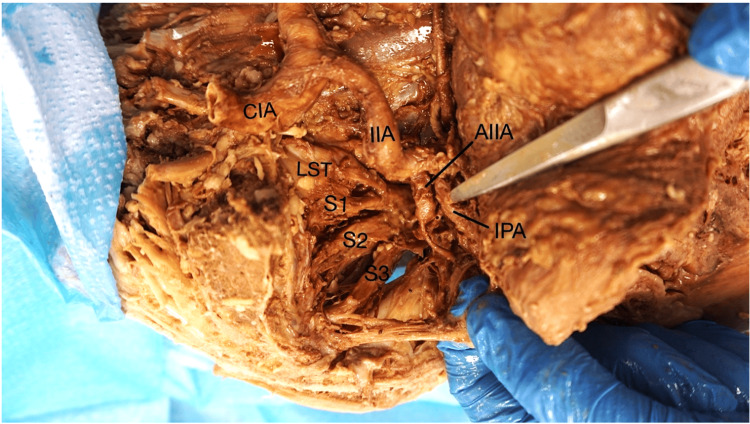
LST and sciatic nerve (SN) roots The LST combines with the first three sacral nerve roots (S1, S2, and S3) to form the beginning of the SN. Also included in this image for reference are the common iliac artery, internal iliac artery, anterior internal iliac artery, and the internal pudendal artery. LST: lumbosacral trunk, CIA: common iliac artery, IIA: internal iliac artery, AIIA: anterior division of internal iliac artery, IPA: internal pudendal artery

**Figure 4:**
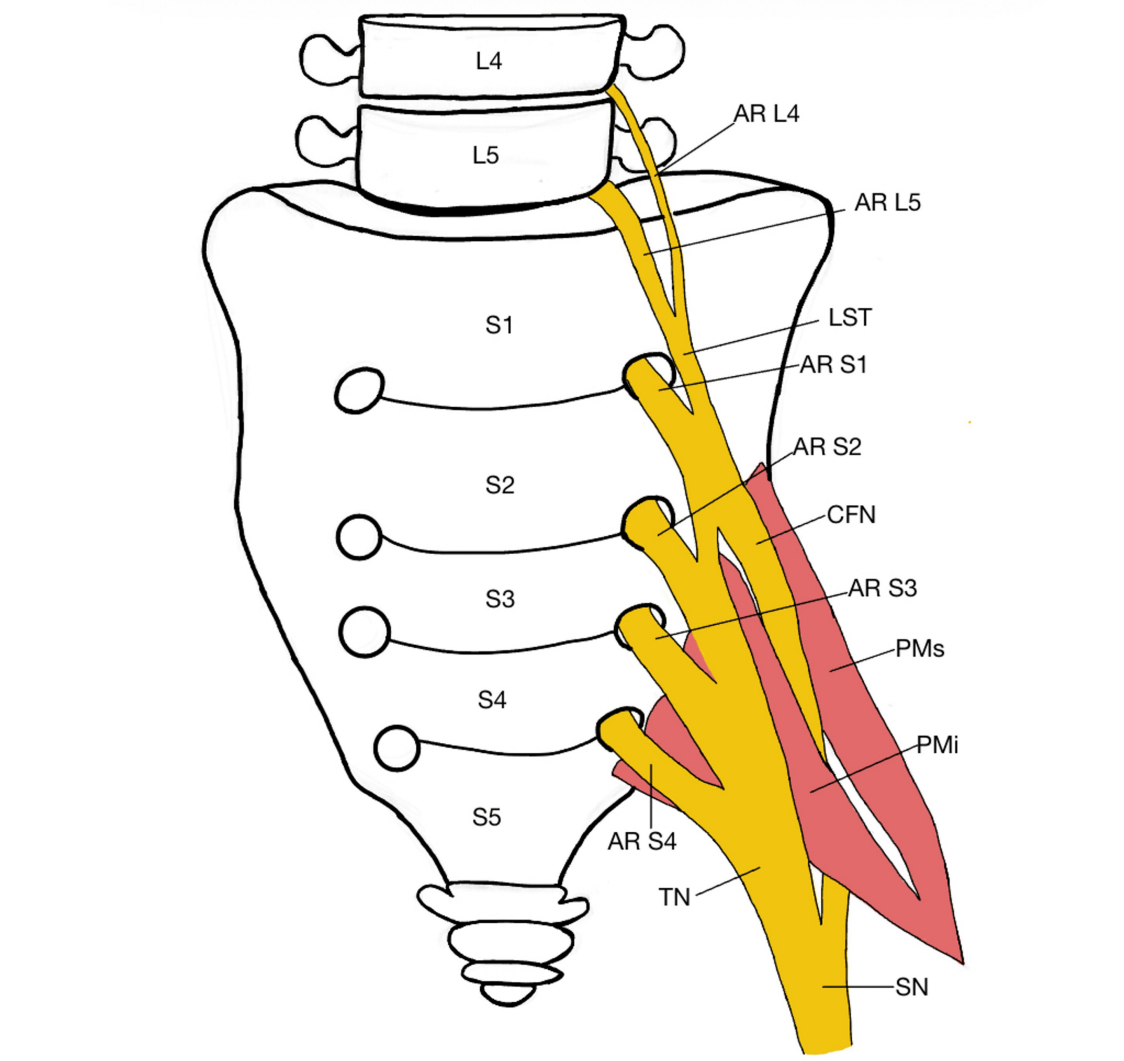
Schematic of contributing nerve roots to sciatic nerve (SN) anomaly The LST combines with contributions from the anterior ramus of S1 to form the CFN, which pierces the PM, forming a superior PM muscle belly and an inferior PM belly. Contributions from the anterior rami of S1-S3 and a minor contribution from the LST combine to form the TN, which dives inferior to the PM to enter the posterior compartment of the thigh. The sciatic nerve forms inferior to the PM. LST: lumbosacral trunk, AR S1: anterior ramus of S1 nerve root, AR S2: anterior ramus of S2 nerve root, AR S3: anterior ramus of S3 nerve root, AR S4: anteror ramus of S4 nerve root, CFN: common fibular nerve, TN: tibial nerve, L4: fourth lumbar vertebrae, L5: fifth lumbar vertebrae, S1: first sacral vertebrae, S2: second sacral vertebrae, S3: third sacral vertebrae, S4: fourth sacral vertebrae, S5: fifth sacral vertebrae, PMs: superior belly of piriformis muscle, PMi: inferior belly of piriformis muscle Source: This image was made by Camille Reaux using Notability (Ginger Labs, San Francisco, CA, USA).

Further dissection of the pelvic floor musculature and sacral plexus revealed an atypical pattern of SN formation. The LST and anterior ramus of S1 united to form the CFN, which then traversed and divided the PM into two heads. In contrast, the TN was formed primarily from the anterior rami of S1-S3, with a minor contribution from the LST, and exited the greater sciatic foramen inferior to the PM (Figures 3, 4).

**Figure 5:**
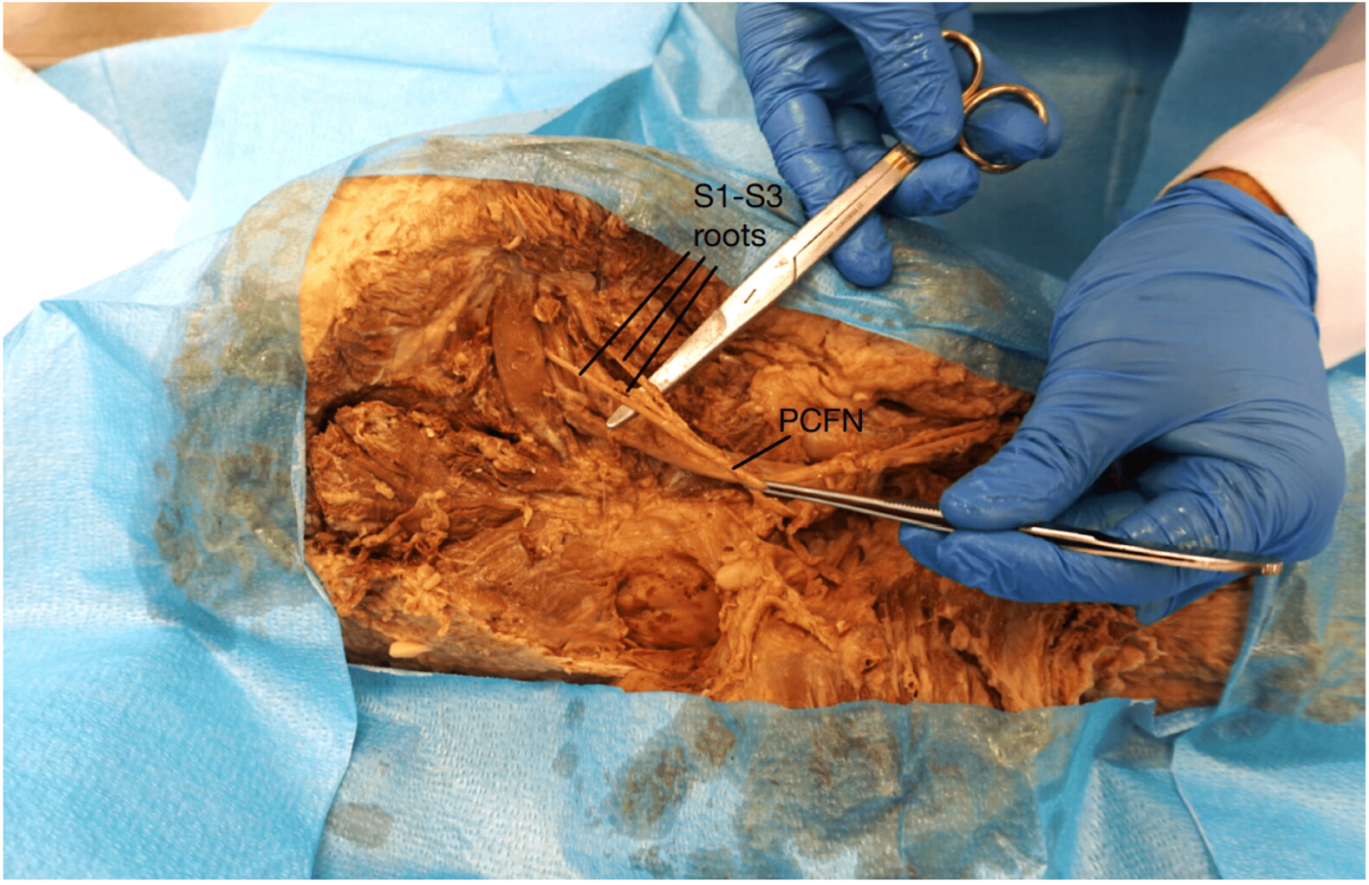
Posterior femoral cutaneous nerve (PFCN) anomaly The PFCN exits the pelvis in its contributory roots. The most lateral root pierces the PM, whereas the two medial roots exit the pelvis deep to the PM. The PFCN is formed 2 inches inferior to the PM. S1-S3 roots: contributory nerve roots (S1-S3) to PFCN

The PFCN was found to exit the greater sciatic foramen in its separate nerve roots, with the most lateral root piercing the PM just medial to the CFN. Its more medial roots exited the pelvis deep to the PM (Figure 5).

## Discussion

In this case report, we presented a unilateral type 2 Beaton and Anson SN anomaly and a PFCN anomaly. The LST and S1 form the CFN, which passes between two heads of the PM. The TN is also formed before its parent nerve, made up of S1-S3, and a small contribution from the LST, but passes beneath both heads of the PM. The TN and CFN form the SN below the PM. The split PM has two heads with one common insertion. The SN is associated with clinical complaints in its typical anatomy and in cases of variation [9]. A common clinical manifestation of SN irritation is sciatica, radiating leg pain below the knee [10]. More of a symptom than a diagnosis, sciatica can stem from many causes, such as disc herniation, a spinal cause, or nerve compression, a non-spinal cause [11]. Sciatica can refer to a host of complications such as non-specific low back pain, radiating nerve pain, or dermatomal paresthesia [11]. The National Interview Health Survey, conducted in 2019, found that 8.2% of US adults live with chronic severe back pain. These patients experience greater disability when their pain is not appropriately managed, highlighting the importance of understanding each individual's etiology of pain to guide effective treatment [12]. Lower back pain can greatly impact a patient’s quality of life by diminishing one’s daily living activities, ability to work, and sexual function [13].

Symptomatic compression of the SN by the PM is known as PS, which is under the branch of non-spinal causes of sciatica. Patients describe their symptoms as buttock pain that radiates along the back of their leg. The pain is often accompanied by numbness or a pins-and-needles sensation. This syndrome lacks a definitive gold-standard diagnostic test, which can delay appropriate treatment for patients. Traditionally, it’s been done through physical exam maneuvers like straight leg raise (patients lies supine as the symptomatic leg is raised by flexing at the hip with the knee extended), Freiberg sign (patient lies supine with the symptomatic leg in internal rotation at the hip joint, the patient externally rotates the hip against counterforce), Pace sign (patient lies supine with knees bilaterally flexed, patient abducts their hips against counterforce), and direct palpation, all of which help assess potential sciatic irritation caused by the PM [14]. If these maneuvers reproduce symptoms, they are considered positive tests. MRI can be used to visualize the size of the PM, since hypertrophy is associated with symptoms, but not to definitely diagnose PS [15]. Ultrasound, on the other hand, is emerging as a reliable and convenient option, providing real-time visualization of the PM and SN interaction [16]. Moving forward, credible research points to optimizing ultrasound as a diagnostic tool for PS, potentially making it a go-to method.

Recognizing and understanding anatomical variations are essential for not only minimizing iatrogenic injury, especially during procedures such as bone cancer biopsy and hip arthroplasty, but also for the treatment of chronic pain and SN irritation [17,8]. Chronic pain can negatively affect a patient’s life physiologically and mentally, and management of chronic pain is important to improve patient outcomes and foster dynamic healing [8]. Our cadaver presented with a type 2(B) SN anomaly. A review conducted by Smoll concluded that any SN variation that results in part of the SN piercing the piriformis has potential for PS, although there is not a stronger likelihood compared to normal anatomy [6]. Contrastingly, in a retrospective review conducted by Khan et al., 41.9% of the participants with a type 2 SN variation were diagnosed with sciatica, suggesting there may be an increased risk of developing sciatica with a type 2 variation [18]. Previous research points towards an increased prevalence of PS in females compared to males. Pace and Nagle found a female-to-male ratio of 6:1 in reviewing 45 cases of PS [19].

We speculate that our patient may have suffered from PS or foot drop at one point in her life, especially if she was physically active, causing piriformis hypertrophy and thus irritation of the CFN and lateral root of her PFCN. The neuropathy caused by PS is hypothesized to be due to the involvement of the PFCN [20]. Given our patient’s nervous system anomalies, it is not unlikely that she experienced PS with PFCN neuropathy at some point in her life. She was also at an increased risk of nerve injury due to the CFN and PFCN lying more superficially than typical anatomy, with procedures such as gluteal injections. Another instance where she would have had an increased risk of nerve injury would be in the case of a proximal hamstring repair, in the case of avulsion or nerve damage from a nerve block procedure. Remy et al. demonstrated the proximity of the PFCN to the surgical field of a proximal hamstring repair, demonstrating that the perennial branch crosses the field transversely [21]. It is important to document nerve anomalies so that clinicians and surgeons have an accurate understanding of anomaly prevalence and thus prepare accordingly prior to a procedure.

If our patient did suffer from PS, identifying the etiology of her pain along with targeted treatment may have improved her quality of life. The treatment of PS varies depending on the root cause of nerve irritation. Effective pharmaceutical approaches include steroid injections and anti-inflammatory medications [5]. Less invasive options exist as well. Osteopathic manipulative medicine (OMM) is a nonpharmaceutical and inexpensive approach to treating PS. OMM techniques that have been found to effectively treat PS include the positional release technique, the muscle energy technique, and a combination of the two prior techniques (integrated neuromuscular inhibition technique) [22,23].

## Conclusions

The early bifurcation of the SN and the late formation of the PFCN represent significant anatomical variations with clinical implications across various fields, including surgery, pain management, rehabilitation, and OMM. In cases of the type 2(B) variant, where the CFN pierces rather than passes beneath the PM, current literature suggests patients face an increased risk of SN entrapment syndromes and are susceptible to iatrogenic injury. This reinforces the importance of thorough, individualized assessments for patients presenting with lower back pain and sciatica-like symptoms. By becoming more aware of these anatomical variants, physicians can ensure safer, more personalized patient-centered care.
